# Supplementary material for: A multi-level analysis of prevalence and factors associated with caesarean section in Nigeria
Source: PLOS Glob Public Health. 2023 Jun 26;3(6):e0000688. doi: 10.1371/journal.pgph.0000688 (PMC10292705; doi:10.1371/journal.pgph.0000688)
Supplement: Attachment — Submitted filename: R&R_comments.docx [file pgph.0000688.s001.docx]

Reviewer #1: The manuscript examined caesarean delivery in Nigeria using individual and community characteristics as the explanatory factors. This is an important public health concern in the country due to the steady rise in the demand for CS despite the poor state of obstetrics and emergency care in the country. The study was well conceptualized but poorly executed. The selection and recoding of the variables need modification. The findings were adequately reported. There are no serious ethical issues raised by the study. I have no evidence that the study is plagiarized work. The writing style of the authors is satisfactory but the manuscript in its current version needs substantial revision. Specific comments on the sections are outlined below:

*We specially thank the reviewer for their thoughtful and comprehensive review of our work to further strengthen the paper. We have carefully responded to the comments and are confident that the paper now meets publication standard. Below we outline our response to the comments and questions raised.*

Abstract

The abstract is informative but the background section needs to show the knowledge gap study intends to fill.

***Response:*** *We have included the verbiage below to show the knowledge gap this study fills.*

*“However, suboptimal CS uptake suggest unmet obstetric care leading to poor maternal and neonatal health. Considering that maternal health problems in Nigeria remains a public health problem, this present study aims to assess the prevalence and multilevel factors associated with CS among women of reproductive age to establish knowledge on CS use on maternal health in the country.” P2 L27-29*

Introduction

a. Authors wrongly claimed that no studies have examined the multilevel factors predicting CS utilization in Nigeria. For instance, see https://doi.org/10.1080/07399332.2018.1443107 and https://doi.org/10.5281/zenodo.6446080. Authors should examine these studies to properly identify a knowledge gap to be filled by their study.

***Response****: We thank the reviewer for bringing these studies to our attention. However, after carefully going through these articles, we also noticed that while Solanke’s (*[*https://doi.org/10.1080/07399332.2018.1443107*](https://doi.org/10.1080/07399332.2018.1443107)*) performed a multilevel analysis, their work was restricted to only one particular region and used data from 2003-2013 Nigeria DHS. while Berglungh and colleague (*[*https://doi.org/10.5281/zenodo.6446080*](https://doi.org/10.5281/zenodo.6446080)*) conducted the regular logistic regression using the 2013 dataset and not a multilevel analysis. However, we reframed the sentence and referenced Solanke’s work. P4 L84. Our work builds on these studies by employing the multilevel analysis to further provide knowledge on the multilevel factors that predict CS uptake in Nigeria using the 2018 Nigeria DHS.*

b. The authors should identify existing policy/strategies that their findings may inform

***Response:*** *We have identified existing policy/strategies that this study findings may inform. See verbiage below:*

*“…For example, programs that promote maternal health care utilization by addressing proximal (e.g., health care seeking attitudes during pregnancy) and distal factors (e.g., education of skilled birth attendants) hold promise to addressing multilevel determinants influencing knowledge and uptake of CS, thereby improving maternal and infant health in Nigeria.” P4 L89-93*

c. The study should be guided by an appropriate research question or hypothesis

***Response:*** *We have included the following research questions.*

*“The study aims to answer the research question: What is the prevalence and the multilevel factors associated with CS delivery in Nigeria?” P5 L95-96*

d. If possible, authors should identify a theory or analytical model that could underpin the study

***Response:*** *Considering that we conducted multilevel analysis, our study aligns with the Newman’s healthcare utilization framework. We have included the following the verbiage.*

*“Overall, this study aligns with the Newman’s healthcare utilization theory that posits that individual (e.g., individual-level factors such as maternal age, ethnicity, parity, religion, child’s size, and antenatal care visits) and societal/communal factors (e.g., household/community-level factors such as place of residence, wealth index, region, and community literacy level) (40). Based on Newman’s theoretical framework, our results indicates that there is a confluence of determinants that are associated with CS uptake in Nigeria, as such, interventions and programs should adopt a holistic multipronged strategy to increase CS uptake in Nigeria to ultimately improve maternal and new born health in Nigeria.” P14-15, L316-323.*

Methods

a) Authors should describe the exclusion criteria

***Response:*** *We have included the inclusion and exclusion criteria. See the verbiage below.*

*“. Only reproductive aged women with at least one child in the last five years were included in this study. The exclusion criteria were women who had no child in the last five years and/or were pregnant for the first time at the time of the survey.” P 6: 115-119*

b) Outcome variable: the authors should describe how the variable was coded to help readers know the category of interest. If possible, authors should cite studies that adopted similar operationalization

*Response: We have reframed this paragraph to help readers understand how the outcome variable was coded and also cited previous studies who adopted similar operationalization.*

*“**Specifically, women responded to questions about whether they gave birth through CS or not. We then coded 0 “No” (indicating that the last birth was non-CS) and 1 “Yes” (indicating that the last birth was via CS).* *Our category of interest was women who responded in the affirmative to having a CS delivery.” P6 L123-126*

c) Individual-level explanatory variable: (i) it does not look good to group secondary and higher education together. Evidence shows that secondary education is the dominant level attained by women in the country. The few that attained higher education may show different socioeconomic status and characteristics (ii) authors should have focused on the major ethnic group of Hausa/Fulani, Igbo, and Yoruba while excluding minority groups. It is offensive to describe separate nationalities as ‘Others’. In addition, this group of ‘others’ has different socio-cultural practices that may affect their perception and utilization of CS (iii) child sex and size should have been control variables because they are not individual characteristics of the women used as a unit of analysis

***Response:***

*We agree that grouping secondary and higher education together may mask the differences in health outcomes. However, we followed the coding scheme of previous studies using the Demographic Health Survey across different health topics and on CS utilization in Nigeria. (See,* [*http://dx.doi.org/10.1136/bmjopen-2018-027273*](http://dx.doi.org/10.1136/bmjopen-2018-027273)*).*

*Regarding the inclusion of “Others,” we agree that combining different heterogenous groups as homogenous may not represent the true characteristics of these groups which might lead to a bias estimate. However, in line with survey data, several reasons such as sample size and other contextual factors may lead to aggregating racial groups into one. In this current study, Hausa, Igbo, and Yoruba are the three major tribes in the country as such it was necessary to include these, while also ensuring people from other groups are represented. As a result, the authors agreed that it is more appropriate to include “others” instead of excluding them because they contain important information about CS use which can influence policy and public health programs. Moreover, previous studies have used this approach (see, 10.4236/oalib.1102747,*

*With child sex and size, we considered these as part of the individual characteristics as they relate to the mother who acts as a respondent on behalf of the child. Additionally, previous studies on CS have controlled for child sex and size (https://doi.org/10.21203/rs.3.rs-1386764/v1 and* [*http://dx.doi.org/10.1136/bmjopen-2018-027273*](http://dx.doi.org/10.1136/bmjopen-2018-027273)*).*

d) Community-level explanatory variables: (i) household head sex is not a community characteristic. Attention should be paid to the fact that individuals are nested in households while households are nested in the community

***Response:***

*Thank you for this comment:*

*“Household” has been added to the community level to show that household variable was included. Head of household was included as a household variable. This change is reflected throughout the manuscript.*

e) Data analysis: this section should describe how the importance of the community-level characteristics was estimated

***Response:***

*Information about the importance of estimating community level characteristics has been included. Page 8, line 171-183.*

f) Describe how variables were selected for the multilevel model. Also, describe the essence of each model (what do they control for?)

***Response:***

*The importance of each model has been explained in Page 8, line 171-183.*

Results

a. As shown in Table 2, why are household wealth and sex of head of household presented as contextual factors instead of household factors? The two variables were not aggregated

***Response:***

*“Household” has been added to the community level to show that household variable was included. Head of household was included as a household variable. This change is reflected throughout the manuscript.*

b. The reported level of secondary/higher education (67.84%) is bogus because of the way the variable was recoded. I doubt if any study has ever found such in the country.

***Response:***

*As we noted in our earlier comment, concerning grouping secondary and higher education together and considering that we followed the coding scheme of previous studies using the Demographic Health Survey across different health topics and on CS utilization in Nigeria (See,* [*http://dx.doi.org/10.1136/bmjopen-2018-027273*](http://dx.doi.org/10.1136/bmjopen-2018-027273)*), this result is similar to those found in previous studies.*

c. Results of the fixed effects should be presented model-by-model as done for the random effects so that readers can follow the importance of variables being added into successive models. Alternatively, the authors may state in the preceding section that the full model is the main model that will be explained

***Response:***

*Both fixed effects and random effects have been reported model by model in the table 2.*

Discussion

a. Authors suggested the “need to multipronged implement strategies to increase CS delivery and to address barriers that affect its utilization” While I agree that the barriers should be addressed among those who demanded it, I wish to know if increasing CS delivery should be a goal in the absence of a medical recommendation

***Response:***

*Increasing CS delivery is an important lifesaving intervention but high rates may increase maternal risks, overstretched health systems and adversely impact future pregnancy, especially in the absence of medical recommendations (See; Begum et al 10.1371/journal.pone.0188084). However, our study highlighted the need for the inclusion of regional, local and cultural difference contexts for evidence-based policy and programmatic efforts to facilitate equitable access to CS in Nigeria for those in need (See our conclusion). We made this submission because there are many studies (ours inclusive) showing that social, economic, cultural setting factors and even “physician-related factors” which may attribute the rise in CS not to obstetric risk factors. This may are also underlie underutilization rate of CS in many settings, regions, ethnic group and distribution of medical indication (See; Elnakib et al. 10.1186/s12884-019-2558-2, Poma 10.1016/s0002-9378(99)70021-9,Goyert et al. 10.1056/NEMJ19890316201106, DeMott et al. 10.1016/002-9378(90)90925-w). So we advocated that not a size fit all approach should be used, but a multipronged strategy that will address all the barriers raised with recommendations herein to increase CS delivery for those who need it (CS prevalence is 3.1% vs WHO max 10%).*
