# Supplementary material for: A multi-level analysis of prevalence and factors associated with caesarean section in Nigeria
Source: PLOS Glob Public Health. 2023 Jun 26;3(6):e0000688. doi: 10.1371/journal.pgph.0000688 (PMC10292705; doi:10.1371/journal.pgph.0000688)
Supplement: Attachment — Submitted filename: R&R_comments_2.docx [file pgph.0000688.s002.docx]

**Reviewers and comments**

We thank the reviewers for their detailed and constructive feedback and have carefully responded to the comments.

Reviewer #1: 1. The statement on Lines 310-313 is not completed
2. Check the theory mentioned in Line 310. It ought to be Andersen-Newman

Reply

This has been corrected. Page14, Line 363.

Reviewer #2: 1. SUMMARY OF THE ARTICLE:

The study assessed the national prevalence of CS uptake in Nigeria using DHS 2018 cross-sectional data and estimated the main effect of associated factors at the individual and supra-individual levels, adjusted for covariates. Being of Yoruba ethnicity, parity>=2, practicing Islam, “average” birth weight baby, rural residency, and being from the south-south region of Nigeria predicted less likelihood of CS uptake when compared with Hausa women, those with parity=1, Christians, having macrocosmic babies, living in urban areas and the North Central. The authors chose a valid research question of contemporary public health importance to the research setting, and fits within the context of existing literature in related discipline. Hence, the findings of the study will add value to the body of knowledge, which could influence further studies and policy action. The study design, sample size and choice of multi-level analysis are apt but, the conceptual definition, delineation and classification of what constitute the multi-levels and factors in each level was seemingly defective and poorly executed. I will recommend that a major revision of the article with the multi-level analysis repeated before acceptance for publication.

Reply

Thank you for taking time to review our manuscript, all issues raised have been addressed.


2. MAJOR ISSUES:

The study was designed to do a multilevel analysis. As defined by the authors, the individual, household, and community levels are not too many to justify any need to collapse household and community factors together. Theoretically, household factors and community factors are distinct enough to warrant delineation. It will be useful for the authors to show effects of factors at the household level differently from those at the community level, before the final model of all factors.

More importantly, the authors need to meticulously ensure that factors selected in each level are mutually exclusive to avoid multicollinearity. With multicollinearity, the multi-level effect size estimates (regression coefficients) will be biased as estimates of the standard errors and variance inflation factors (VIF) are affected. This is a major problem with the current analysis done by the authors e.g., they selected education, occupation and employment status at the individual level while, wealth index, community literacy level, and community-level of socioeconomic status was selected at the second level. Correlation between these factors is apparent, which implies inherent multicollinearity. Authors should revise the classification of variables at each level and repeat the multi-level model building

Reply

Thank you for the comments. Kindly note that the results of the household/community variables were displayed separately in Table 2 (Model II) and all included variables in the study were combined in (model III) to show the final association while the labelled of the household/community variables in the row-heading was just a label to understand the listed variables underneath and do not depict showing results for the household/community variables as a whole. This statistical approach has been used by numerous scholars with direct link pasted below. However, the relabeling of the table has been done to avoid further confusion.

1. <https://bmcpublichealth.biomedcentral.com/articles/10.1186/s12889-021-10252-7>
2. <https://www.tandfonline.com/doi/full/10.1080/00324728.2021.1892170>
3. <https://journals.plos.org/plosone/article?id=10.1371/journal.pone.0258844>
4. <https://www.hindawi.com/journals/bmri/2021/6696829/>

A similar study conducted under DHS reported same style of modelling. Please check <https://dhsprogram.com/publications/publication-wp104-working-papers.cfm>.

The VIF of the study reported no multicollinearity and a statement has been added to support this. Page 10, lines 214 to 216.

Background:

- Authors needs to highlight in one-two statements, some theoretical framework in literature for the multi-level approach chosen or state how uptake of CS or other related maternal health services have been shown to be determined differently by factors at the various levels. This study is not the first to think this way. The Newman’s healthcare utilization theory mentioned in the discussion should have been mentioned here at first as the background to their chosen approach.

Reply

Thank you for this comment. We have discussed the Andersen healthcare model in the background section. Please see Page 5, line 106-110.

Methods (variable Definition and Data Analysis):

- The authors should consider replacing the term “child size” with the more specific term “child birth weight” because size could imply other anthropometric measures like length/height. Also, birth weight is usually classified as low, normal or high. There is a standard definition of those. The authors used small, average and large without defining what this means, if any different from low, normal & high birth weight

Reply

All “child size” has been changed to “Child birth weight” and the sub-variable has been changed from “large, average and small” to “high normal and low” as advised.

The authors merged Hausa and Fulani into one category. The name of the final category should reflect that it includes Fulani women, and not just Hausa. For the regression analysis, it may be more logical to “others” the reference category, so you can compare the hausa, fulani and igbo to others.

Reply

Fulani has been added to show that it included both similar ethnic group throughout the manuscript. It’s now worded as Hausa/Fulani


3. MINOR ISSUES:

It is a well written article overall but, there are many grammatical and typographical errors throughout the article, which can be easily corrected if the [many] authors will consider proof-reading carefully or have a language expert do that for them.

Reply

All authors have proofread the manuscript and have made significant English proofreading.

The Title: it is unnecessary verbose with no added information of some included/repeated words. E.g., the phrase “among women of reproductive age” can be excluded since CS delivery is typically expected in pregnant women that are often of reproductive age. Moreso that DHS is on the title, and DHS usually collects data from that age-group. Also, Nigeria did not need to appear twice in that title. Consider revising the title.

Reply

The title of the manuscript has been changed to “A multi-level analysis of prevalence and factors associated with Caesarean Section delivery in Nigeria”


The Abstract: Authors should consider revising the entire background statement for grammar, tenses and flow of thought. “Vaginal delivery” mentioned at the beginning has questionable value as the article is entirely about CS delivery. Also, “maternal health” does not include “neonatal health”. Authors should revise that statement. The methods and result section under abstract should be re-written after authors have revised the article and repeated the multi-level analysis. The conclusion should be revised to reflect the specific findings of this study and not what the authors wish, generally.

Reply

Thank you for this comment. We have revised the abstract section as advised. Page 2, L 30 to 57


4. OTHER POINTS (optional notes):

- The tables could be better formatted and improved. Authors can look up similar published tables for guides

Reply

Table has been adjusted and relabeled where necessary.

- The discussion, conclusion and recommendations could be more robust, systematic, and specific to the findings of this study.

Reply

Thank you for this comment. The discussion, conclusion, and recommendations were presented based on the findings. However, we have made edits as needed to make this section more robust and systematic. Pages 14 to 17.

Reviewer #3:

Review of manuscript entitled: “Prevalence and Factors Associated with Caesarean Section Delivery among women of reproductive age in Nigeria: A Multi-level Analysis of the 2018 Nigeria Demographic and Health Survey”

Thank you for the opportunity to review this manuscript. This study uses a multilevel analysis to assess the relationship between variables at the individual level and household/community level and cesarean section deliveries in Nigeria using a nationally representative household survey. The findings of this study provide important insights into the characteristics of women and the households/communities they reside in and how these characteristics differ for women who delivered their last birth as a cesarean section versus normal vaginal delivery. The paper is well-written and clearly presents the results and discusses the findings as well as limitations of the study. Nevertheless, I felt that most of the interpretations of the results in the discussion were targeted towards the women themselves. In reality, it is not the women who should choose whether to have a CS. I believe the importance of the community level indicators is to shed light on the possibility that the quality of care (ANC and obstetrics services) varies and that there should be interventions that target healthcare services and birth attendants as well, not only the women themselves.

Major comments

Discussion:

•	On line 260-261, please consider rewording the sentence: “this study calls for strategies to encourage CS delivery among younger women, particularly those at risk of obstetric complications.” This sentence may be mis-understood to encourage elective CS as well, which is a big problem in many countries. Encouraging awareness of and access to proper access to ANC, quality health-care facilities and skilled birth attendants may send a better message.

Reply

Thank you for this comment. We have reframed this sentence. Please see Page 14, L 294-297

•	When discussing parity and how higher parity is associated with higher CS deliveries (line 286), one reason could be that the women may have had previous CS deliveries. Was this considered in the analysis? Another explanation for this finding is that women who have higher parity are also older, hence are at higher risk of developing complications that require more medical interventions and CS deliveries.

Reply

Thank you for this comment. The data does not ask questions about previous CS delivery. However, we have reframed this sentence to include the insights recommended. See Page 16, lines 333-335.

Strengths and limitations:

•	Another strength of this study that may be added to this section, is the use of multi-level analysis to explore individual and household/community level.

Reply

This statement has been added. Page 17, lines 371-373

•	With regards to the limitations, you may want to include a justification for not considering the variables related to place of delivery and whether the birth attendant was skilled or unskilled.

Reply

Justification has been provided. Page 18, lines 382-384

Minor comments

Abstract

•	On line 30, the word Nigeria is missing: “…factors associated with CS among women of reproductive age in to establish knowledge on CS use on maternal health in the country.

Reply

This has been included. Page 2, line 35

•	The start of the sentence on line 47 is a bit unlcear: “Our study highlights the need for the inclusion of regional, local, and cultural difference contexts”. I would suggest rewording it to: “Our study highlights the need for the inclusion of different regional, local, and cultural contexts”.

Reply

Thank you for this comment. We have reworded this section as advised. Please see page 3, Line 56.

Background

•	Reference number 1 cited on line 52 may be updated to a newer publication for the same other with more recent CS trends from 2018 (“Trends and projections of caesarean section rates: global and regional estimates” by Betran et al doi - http://dx.doi.org/10.1136/bmjgh-2021-005671

Reply

Thank you for sharing the updated reference. Page 20, line 421 -424

•	The sentence in line 57-58 requires editing to become: “More specifically, the prevalence of CS in Africa and sub-Saharan Africa is 7.3% and 4.9%, respectively in sub-Saharan Africa”.

Reply

Thank you. We have reworded the sentence. Page 4, line 70-71

•	On line 67, the phrase “…all of which are higher than other most African countries” should be corrected to “all of which are higher than most of the other African countries”.

Reply

Thank you. We have reworded the sentence. Page 4, line 82-83

•	On line 69, the word ‘mortality’ is missing in the phrase “seeks to reduce the global maternal ratio”

Reply

Thank you. We have reworded the sentence. Page 4, line 85

•	On line 80, ‘place of residence’ may be better than ‘residence’

Reply

Thank you. We have reworded the sentence. Page 5, line 98

Methods and materials

•	On line 121, the word ‘specifically’ is repeated twice.

Reply

Thank you. This has been corrected. Page 7, line 148

•	On line 162, the sentence “We computed the weighted summary statistics of socioeconomic and demographic variables stratified by the dependent variable (caesarean delivery)” should be consistent with the rest of the paragraph: “The weighted summary statistics of socioeconomic and demographic variables were computed and stratified by the dependent variable (caesarean delivery).”

Reply

This has been corrected. Page 11, lines 191 to 192

Results

•	On line 198, when referring to the ANC visits, please mention four or more visits.

Reply

Thank you. This has been corrected. Page 11, L 238

•	On line 200, to stay consistent throughout the text, it may be better to say “at the household/community level…”

Reply

“Household” has been added to the statement to ensure consistency. Page 12, line 240.

•	On line 201, the sentence should read: “…and males headed 18,590 households (93.11%)” rather than females headed this number of households.

Reply

Thank you. This has been corrected. Page 12, line 241

•	In the paragraph from line 210 to 213, please add units to the age of women.

Reply

Thank you. This has been corrected. Page 13, line 254

•	On line 219, please remove ‘whose’.

Reply

Thank you. This has been corrected. Page 13, line 260

•	On line 238, is it supposed to be incomplete or complete model?

Reply

This has been corrected. Page 13, Line 280

•	The title of table 1, on line 488, “Table 1: Individual & household/conmmunity factors of respondents by C-section delivery in Nigeria” requires some revision. The word ‘community’ is mis-spelt. I would suggest a more comprehensive title: “Table 1: Distribution of individual & household/community factors among women aged 15-45 years old and prevalence of cesarean section delivery in Nigeria, NDHS 2018.”

Reply

This has been corrected. Check table 2 on page 26.

•	The title of table 2 on line 446: “Table 2: Multilevel logistic regression models for individual and contextual factors associated Caesarean Section Delivery in Nigeria” may be reworded to “Table 2: Multilevel logistic regression models for individual and household/community factors associated with Caesarean section deliveries among women aged 15-45 in Nigeria, NDHS 2018”

Reply

This has been corrected. Check table 2 on page 31

Discussion

•	In the first paragraph of the discussion, there is a mixture of past and present tense. The present tense needs to be changed to past tense.

Reply

Thank you. This has been corrected. Page 14, line 290-298

•	On line 253, the phrase ‘need to multipronged implement strategies’ is unclear. Please reword it.

Reply

Thank you. We have reworded this sentence. Page 14, line 296-298

•	On line 256, the multi-level model numbers need to be converted into Roman numerals. Also, I believe it should be models I and III not I and II.

Reply

This comment is not clear. However, the study developed three models (Model I, Model II, and Model III). All these models’ results were interpreted in line with the findings under each model. The numbers I,II, and III are roman figures.

•	The sentence on line 310-313 seems to be incomplete, please consider revising it.

Reply

Thank you. This has been corrected. Page 16, line 328-330

Conclusion

•	Similar to the abstract, on line 334 the sentence is a bit unclear: “it is clear that there is a need for the inclusion of regional, local, and cultural difference contexts for evidence-based policy and programmatic efforts”. I would suggest rewording it to: “…for the inclusion of different regional, local, and cultural contexts”.

Reply

Thank you. This has been corrected. Page 19, line 392-393
