# Supplementary material for: A multi-level analysis of prevalence and factors associated with caesarean section in Nigeria
Source: PLOS Glob Public Health. 2023 Jun 26;3(6):e0000688. doi: 10.1371/journal.pgph.0000688 (PMC10292705; doi:10.1371/journal.pgph.0000688)
Supplement: Attachment — Submitted filename: Comments and Replies_R4.docx [file pgph.0000688.s003.docx]

**Comments and Replies**

Major issues:

The model building strategy could be slightly clearer and systematic. How were variables added to the model and how did you chose if they were included in the final model?

There is a good summary on model selection techniques: Laubach, Zachary M., et al. "A biologist's guide to model selection and causal inference." Proceedings of the Royal Society B 288.1943 (2021): 20202815. [Pre-print version (open access): <https://arxiv.org/ftp/arxiv/papers/2010/2010.07506.pdf>]

Reply

We have added the condition behind the selection of the variables into the multilevel model. Page 9, Line 186 to 187.

Ultimately - what is the aim of the model building strategy is it to obtain a predictive model (identify a set of variables that best explain the variation in the outcome) or is it to understand the relationship between each factor and the outcome? If it the latter each exposure should be modelled separately with the outcome due to the table 2 fallacy:

Westreich, Daniel, and Sander Greenland. "The table 2 fallacy: presenting and interpreting confounder and modifier coefficients." American journal of epidemiology 177.4 (2013): 292-298.

If it is to build a predictive model then the PROBAST, TRIPOD guidelines require to be followed. Also, the interpretation of the adjusted coefficients requires to be removed as they are not interpretable due to the table 2 fallacy.

Reply

The aimed of the study was to better understand the relationship between the selected exposure and outcome variables, therefore, the included explanatory variables at each level of the analysis has been remodelled seperately (Individual, household, and community), at the sametime we still remained the last model (Model IV) to show the relationship of all the explanatory variables together on the outcomes variable. See table two of the revised manuscript.

Minor changes required:

Abstract: remove the word predict and the statistical test from the conclusion. Rather try and conclude about what the results show?

Reply

The abstract section has been rewritten as advised. Page 3, lines 50 to 55.

Throughout I suggest to use the term "use of casearean section" rather than cesearean section rates - as technically cs rate is not a rate although it is prevalent in the literature.

Reply

“use of casearean section" has been used appropritely where applicable throughout the revised manuscript.

Since you are publishing in PLOS there is no word count so remove the abbreviation CS through and replace with unabbreviated form.

Reply

The abstract section has been rewritten as advised. Page 2 to 3, lines 27 to 55.

Line 126 "Specifically, for this study, we used the “KR file” of the 2018 NDHS as recommended by analysts of the Demographic Health Survey" No need to mention the KR - we will assume you have used the correct file.

Reply

This statement has been removed as advised. Page 6, lines 130 to 131.

Line 137-143 - is this C-S at last birth?

Reply

We only included reproductive aged women with at least one child and/or had any child delivery in the last five years in this study. To give persective to those who have had CS and those who have not had CS. More information has been provided in the revised manuscript to avoid confusion. Page 7, lines 135 to 137.

Line 157-161: For the score you created what was the cohen's kappa?

Reply

The score was “nominal” just to label the media exposure.

Line 201: What number did you consider as collinear?

Reply

Full details of the VIF has been included. Page 10, lines 211 to 212.

First paragraph of the discussion should present the main findings of the study only. Then the proceeding paragraph should place the findings in context.

Reply

The first paragraphy of the study discussion section now contains the study main findings as advised. Page 27, lines 306 to 309.
