# Supplementary material for: A multi-level analysis of prevalence and factors associated with caesarean section in Nigeria
Source: PLOS Glob Public Health. 2023 Jun 26;3(6):e0000688. doi: 10.1371/journal.pgph.0000688 (PMC10292705; doi:10.1371/journal.pgph.0000688)
Supplement: Attachment — Submitted filename: Comments and Replies_R5.docx [file pgph.0000688.s004.docx]

**Review comments and replies.**

Reviewer #2: The manuscript assessed the prevalence of CS uptake in Nigeria using DHS 2018 data and attempted to determine the multilevel factors associated with CS uptake. The authors chose a valid research question of contemporary public health importance to the research setting and fits within the context of existing literature in related discipline. Hence, the findings of the study will add value to the body of knowledge, which could influence further studies and policy action. The study design, sample size and choice of multi-level analysis are apt.

The authors made adequate attempt at resolving most of the concerns raised in revision 2, except for their choosing not to disaggregate household and community factors. Considering the current state of the article, I will recommend its acceptance for publication, provided that the authors are made to correct the typographical errors that persist in the paper. Also, they should consider reformatting the tables.

Reply

Thank you for taking the time to review our manuscript again. We have reformatted the tables, and we have ensured that the tables are easy to read. We have also proofread the manuscript for language editing.

Reviewer #3: Review of manuscript entitled: “A multi-level analysis of prevalence and factors associated with Caesarean Section delivery in Nigeria”

Thank you for the opportunity to review this manuscript again. This study uses a multilevel analysis to assess the relationship between variables at the individual level and household/community level and cesarean section deliveries in Nigeria using a nationally representative household survey. The findings of this study provide important insights into the characteristics of women and the households/communities they reside in and how these characteristics differ for women who delivered their last birth as a cesarean section versus normal vaginal delivery.

The authors have attended to the reviewer’s comments and this version reads very well. The manuscript clearly presents the results and discusses the findings in a comprehensive way. The limitations of the study are well articulated.

Some comments, mainly minor, are listed below:

• On p.16, the results section “Random effects” starts by referring to the variation based on the clustering level of the MLM. And then you change to referring to the ICC. You interchange between the terms variation and ICC and I think it should be written in a clearer way.

Reply

Thank you for taking the time to review our manuscript again. This has been rewritten to ensure it’s clear enough. Please see page 16 to 17 of the revised manuscript.

• With regards to the ICC, it would be useful to give a brief description in the methodology about how you estimated this value since in logistic MLMs it is not directly possible to generate an ICC.

Reply

More information on ICC has been added on pages 10 to 11 of the revised manuscript.

• On p.9, line 186: there is a typo with regards to the value of the p-value.

• On p.9, line 191: the sentence wording needs to be revisited.

• On p.26, line 299 there is another typo in the sentence.

Reply

All typo within the manuscript has been corrected, and the manuscript has been reviewed for any other possible English language error.
